# Modified Frailty Index to Assess Risk in Elderly Patients Undergoing Distal Pancreatectomy: A Retrospective Single-Center Study

**DOI:** 10.1007/s00268-021-06436-2

**Published:** 2022-01-13

**Authors:** Salvatore Paiella, Matteo De Pastena, Alessandro Esposito, Erica Secchettin, Luca Casetti, Giuseppe Malleo, Greta Montagnini, Elisa Bannone, Giacomo Deiro, Beatrice Bampa, Marco Ramera, Luca Landoni, Alberto Balduzzi, Claudio Bassi, Roberto Salvia

**Affiliations:** 1 General and Pancreatic Surgery Unit, Pancreas Institute, University of Verona Hospital, Verona, Italy; 2 Referent of the Mini-Invasive Pancreatic Laparoscopic and Robotic Surgery of the General and Pancreatic Surgery Unit, Department of General and Pancreatic Surgery, The Pancreas Institute, University of Verona Hospital Trust, P.le Scuro 10, 37134 Verona, Italy

## Abstract

**Background:**

To compare the postoperative course of elderly patients (≥70 years) submitted to minimally invasive (MIDP) versus open distal pancreatectomy (ODP) and to evaluate if the modified Frailty Index (mFI) predicts the surgical course of elderly patients submitted to DP.

**Methods:**

Data of patients aged ≥70 who underwent DP at a single institution between March 2011 and December 2019 were retrospectively retrieved. A 2:1 propensity score matching (PSM) was used to correct for differences in baseline characteristics. Then, postoperative complications were compared between the two groups (MIDP vs. ODP). Additionally, the entire cohort of DP elderly patients was stratified according to the mFI into three groups: non-frail (mFI = 0), mildly frail (mFI = 1/2), or severely frail (mFI = 3) and then compared.

**Results:**

A total of 204 patients were analyzed. After PSM, 40 MIDP and 80 ODP patients were identified. The complications considered stratified homogenously between the two groups, with no statistically significant differences. The severity of the postoperative course increased as mFI did among the three groups regarding any complication (*p* = 0.022), abdominal collection (*p* = 0.014), pulmonary complication (*p* = 0.001), postoperative confusion (*p* = 0.047), Clavien-Dindo severity ≥3 events (*p* = 0.036), and length of stay (*p* = 0.018).

**Conclusions:**

Elderly patients can be safely submitted to MIDP. The mFI identifies frail elderly patients more prone to develop surgical and non-surgical complications after DP.

**Supplementary Information:**

The online version contains supplementary material available at 10.1007/s00268-021-06436-2.

## Introduction

Minimally invasive distal pancreatectomy (MIDP) is gaining favor over open distal pancreatectomy (ODP) to treat the pancreas' body-tail neoplasms [1]. An international cohort study showed less major morbidity after MIDP, with predicted better outcomes as the conversion rate decreases following the implementation of the technique [2, 3].

Thanks to intervention in health, political, and socioeconomic aspects, *lifespan,* and *healthspan* are expected to increase in the next decades [4, 5], at least in developed countries. This scenario and the incidence of pancreatic tumors that increase with age–with a mean age of 70 years [5]—are responsible for the frequent facing with elderly patients by pancreatic surgeons. Current literature reports controversial results regarding the impact of advanced age on surgical outcomes after pancreatic surgery. In 2012, a meta-analysis reported that elderly patients experience higher rates of postoperative complications and mortality [6]. Other manuscripts, afterward, found that pancreatic surgery has similar safety and efficacy profiles in elderly versus non-elderly [7–10]. Regarding minimally invasive pancreaticoduodenectomy, data analysis on 1768 elderly patients submitted to laparoscopic versus open pancreaticoduodenectomy from the United States National Cancer Dabatase showed that minimally invasive cases experience lower mortality rates [11]. Few manuscripts have compared MIDP versus ODP in elderly patients. In particular, four retrospective, non-case-matched studies, enrolling a total of 248 patients, reported that MIDP has the same safety profile that ODP in elderly patients [12–14] and that some postoperative outcomes, such as confusion, length of stay, and intraoperative blood loss, are even better for MIDP [15]. Certainly, there is a proper patient selection beyond these surgical cohorts, so maybe the fittest ones were chosen. A recent meta-analysis pooled the results of these studies and confirmed the conclusions, indicating not only that MIDP is not contraindicated in the elderly but even that some postoperative outcomes are better in this subset of patients [16].

Not all elderly patients are the same, and to offer the same surgical approach to each one would be wrong. In particular, the selection of patients is of utmost importance to optimize surgical and oncological outcomes. Several indexes have been elaborated to predict poor postoperative outcomes in elderly patients undergoing pancreatectomy. The Frailty Index [17, 18], and its simplified form, the modified Frailty Index (mFI) [19] proved to predict major complications and mortality after pancreatic surgery effectively [20, 21]. Of note, it seems that frail patients (mFI ≥1) benefit from MIDP, experiencing less severe complications than ODP patients [22].

This study aimed to compare surgical outcomes between MIDP and ODP in elderly patients using propensity-score matching analysis. Secondarily, the efficacy of mFI in predicting postoperative outcomes of DP elderly patients was evaluated.

## Methods

### Study population

The index population for this study was obtained from the electronic Institutional prospectively maintained database of patients submitted to pancreatic resection at the General and Pancreatic Surgery Unit of the Pancreas Institute of the University of Verona, Verona, Italy. The database was queried for patients ≥70 years of age submitted to MIDP (index population), either laparoscopic and robot-assisted, with or without splenectomy, for any disease from March 2011 to December 2019. The database was then queried for patients ≥70 years who underwent ODP (control group) during the same period.

### Data collected

Demographic, clinical, surgical, pathological, and postoperative data were extracted. Demographic and clinical variables included age, gender, smoking habit, body mass index (BMI), albumin, hemoglobin, and leukocyte values, presence of diabetes or other relevant comorbidities, American Society of Anesthesiologist Physical Status Classification System (ASA) score, Charlson Age Comorbidity index (CACI) [23]. When the final diagnosis was of malignancy, Ca 19–9 values and neoadjuvant therapy were added. According to the mFI, patients were stratified into non-frail (mFI = 0), mildly frail (mFI = 1–2), or severely-frail (mFI > 2), as proposed by Konstantinidis et al. [22]. This stratification was applied to the whole cohort of patients ≥70 years who underwent distal pancreatectomy, and matching was not used due to the small sample size of the MIDP group.

Intraoperative variables included intraoperative blood loss (mL), duration of surgery (min), additional resections (yes/no), conversion to ODP, level of pancreatic transection (pancreatic neck/ gastroduodenal artery level/left aortic border), transection technique (stapler/ultrasonic device/ handsewn), splenic preservation.

Ninety-day postoperative surgical data included pancreatic fistula (POPF) [24], delayed gastric emptying (DGE) [25], post-pancreatectomy hemorrhage (PPH) [26], infected abdominal collections treated with percutaneous drainage or antibiotic therapy, chyle leak [27], surgical site infections (SSI), pulmonary or cardiologic complications, and postoperative confusion. Postoperative pathologic data included final diagnosis, tumor diameter, lymph nodes harvested, lymph node status and the number of metastatic nodes (if any), and R-status.

### Surgical procedures

For the description of the Institutional laparoscopic and robot-assisted techniques adopted, previously published material can be considered [28–30]. The decision to submit patients to MIDP and the choice of the laparoscopic or the robot-assisted approach was mostly made on a collegial basis at a dedicated preoperative surgical meeting [31]. The level of transection was made based on the presumed diagnosis (at the pancreatic neck for malignant or intermediate-malignant lesion vs. at the projection of the left border of the aorta for benign lesions, when tumor dimensions allowed it). Regarding the technique of transection, two devices were adopted in the vast majority of cases; the triple row stapler reinforced with a PGA felt (NEOVEIL Endo GIA Reinforced Reload with Tri-Staple® Technology 60 mm, Covidien, North Haven, CT, USA), or an ultrasonic scalpel [32, 33]. Typically, for ODP, two Penrose-type capillary drains were placed at the end of the surgery, one close to the pancreatic stump, the other in the splenic fossa. After MIDP, one Penrose-type capillary drain was placed, close to the pancreatic stump and passing through the splenic fossa. When the patient was enrolled in the DIPLOMA trial (MIDP vs. ODP in patients with pancreatic cancer, #ISRCTN44897265), two drains were placed, one to the left into the splenic fossa and one to the right close to the pancreatic stump. Early drains removal was frequently performed based on the quality and quantity of the output, its richness in amylase, and the occurrence of postoperative acute pancreatitis [34]. Generally, in the presence of a non-amylase-rich fluid (e.g., ≤ 2000 U/L) and the absence of a sinister appearance or postoperative acute pancreatitis, the removal was performed on postoperative day three.

### Statistical analysis and case-matching

According to the normality tests, values were expressed as median and interquartile range, mean and standard deviation (SD), or percentage. Student’s t-test, Mann- Whitney U-test, Kruskal–Wallis test, and *χ*^2^ test were used as appropriate.

A PSM analysis was performed using the preliminary univariate analysis on the entire cohort. The not balanced variables identified were chosen to compare the MIDP (index) group and the ODP (control) group. The MIDP elderly patients were randomly matched with ODP elderly patients according to a 1:2 matching, with a 0.1 caliper and an average treatment effect (ATE), by the approach (MIDP vs. ODP) as the independent variable. Variables considered for propensity score estimation included: age, CACI, splenectomy, additional resections, pancreatic transection level, management of the pancreatic stump, and presence of malignancy at the final pathology. Balance on covariates between MIDP and ODP groups was assessed and reported using absolute standardized differences (ASD) [35]. An ASD value <0.2 indicates a small difference between the two groups, identifying an excellent balance. An ASD value between 0.2 and 0.5 shows a difference, implying a good balance. An ASD value between 0.5 and 0.8 indicates a high difference, meaning sub-optimal balance. An ASD value > 0.8 resulted in a remarkable difference, suggesting a poor balance between the two groups (Supplementary Fig. 1) [36]. Statistical analyses were performed using SPSS software ver. 22 (IBM, Chicago, IL, United States) and R software (R Foundation for Statistical Computing, Vienna, Austria).

## Results

A total of 204 patients aged ≥70 years was submitted to distal pancreatectomy during the study period. Forty-six patients were candidates to receive MIDP, and four (7.7%) were converted to open surgery. The preoperative, intraoperative, and pathological data are shown in Table 1. The two groups resulted imbalanced in age, CACI, splenectomy, additional resections, pancreatic transection level, management of the pancreatic stump, and presence of malignancy at the final pathology (*p* < 0.05).

**Table 1:** Preoperative, intraoperative, and pathological data

Study Population N° = 204				
	Total n° (%)	Open DP 162 (79%)	MI-DP 42 (21%)	*p*-value
*Preoperative data*				
Age (years, DS)	74 ± 4	75 ± 4	74 ± 4	**0.032**
Sex (Female)	108 (53%)	87 (54%)	21 (50%)	0.399
BMI (Kg/m^2^, DS)	25 ± 4	25 ± 4	25 ± 4	0.567
Previous abdominal surgery	114 (56%)	92 (57%)	22 (52%)	0.351
ASA score > III	61 (30%)	53 (33%)	8 (19%)	0.059
Charlson Age > 4	78 (38%)	68 (42%)	10 (24%)	**0.022**
Neoadjuvant therapy	30 (15%)	25 (15%)	5 (12%)	0.383
Frailty index > 0	150 (74%)	120 (74%)	30 (71%)	0.433
*Intraoperative data*				
Spleen preserving	11 (5%)	5 (3%)	6 (14%)	**0.011**
Additional resections	68 (33%)	59 (36%)	9 (21%)	**0.046**
Vascular resections	31 (15%)	28 (17%)	3 (7%)	0.076
Transection level				** <0.001**
Pancreatic neck	165 (81%)	130 (80%)	35 (83%)	
GDA level	29 (14%)	29 (18%)	0 (0%)	
Left aortic border	10 (5%)	3 (2%)	7 (17%)	
Management Stump				**0.001**
Stapler	85 (44%)	67 (44%)	18 (43%)	
Ultrasonic scalpel	64 (33%)	42 (28%)	22 (52%)	
Handsewn	45 (23%)	43 (28%)	2 (5%)	
Duration of Surgery (minutes, DS)	260 ± 93	258 ± 88	267 ± 95	0.175
EBL (cc, IQR)	200 [100–400]	250 [100–400]	200 [150–300]	0.998
*Pathological data*				
Final Pathology, No. (%)				** <0.001**
PDAC	119 (58%)	102 (63%)	17 (41%)	
pNET	33 (16%)	16 (10%)	17 (40%)	
IPMN	27 (13%)	23 (14%)	4 (10%)	
MCN/SCN	11 (6%)	8 (5%)	3 (7%)	
Other	14 (7%)	13 (8%)	1 (2%)	
Tumor Size (mm, IQR)	29 [20–45]	30 [20–45]	26 [18–40]	0.372
Harvest Lymph nodes (IQR)	28 [19–37]	32 [23–40]	24 [13–33]	**0.001**
R0 Status	159 (81%)	125 (80%)	34 (81%)	0.300

Bold values indicate statistical significance at the *p* < 0.05 level

*BMI* Body mass index; *ASA* American society of Anesthesiology; *EBL* Estimated blood loss; *PDAC* Pancreatic ductal adenocarcinoma; *pNET* pancreatic neuroendocrine tumor; *IPMN* Intraductal pancreatic mucinous neoplasm; *MCN* Mucinous cystic neoplasm; *SCN* Serous cystic neoplasm

The postoperative outcomes of the series are outlined in Table 2. The postoperative clinical course of the entire cohort was comparable (*p* > 0.05). Notably, the ODP group had a higher mean length of stay than the MIDP (15 vs. 10 days, respectively, *p* = 0.042).

**Table 2:** Postoperative data

Study Population N° = 204				
	Total n° (%)	Open DP 162 (79%)	MI-DP 42 (21%)	*p*-value
Any complications	118 (58%)	93 (57%)	25 (60%)	0.473
POPF	38 (19%)	28 (17%)	10 (24%)	0.204
Abdominal collections	70 (34%)	53 (33%)	17 (41%)	0.222
DGE	5 (3%)	5 (3%)	0 (0%)	0.312
PPH	25 (12%)	20 (12%)	5 (12%)	0.589
SSI	18 (9%)	15 (9%)	3 (7%)	0.470
Pulmonary complications	53 (26%)	43 (27%)	10 (24%)	0.443
Cardiovascular complications	18 (9%)	17 (11%)	1 (2%)	0.080
Postoperative Confusion	8 (4%)	8 (5%)	0 (0%)	0.152
ICU Admission	17 (8%)	15 (9%)	2 (5%)	0.274
Clavien-Dindo > 3	27 (13%)	22 (14%)	5 (12%)	0.503
Length of Stay (days, DS)	13 ± 6	15 ± 6	10 ± 7	**0.042**
Reoperations	11 (5%)	8 (5%)	3 (7%)	0.403
Readmissions	14 (7%)	10 (6%)	4 (9%)	0.323
Mortality	2 (1%)	2 (1%)	0 (0%)	0.628

Bold value indicates statistical significance at the *p* < 0.05 level

*POPF* Postoperative pancreatic fistula; *DGE* Delayed gastric empty; *PPH* Post pancreatectomy hemorrhage; *ICU* Intensive care unit

After matching, 40 and 80 patients constituted the MIDP group and ODP group, respectively.

### MIDP versus ODP after matching: baseline, surgical, pathology, and postoperative results

No differences were found in the patients’ baseline characteristics between MIDP and ODP groups after propensity score weighting was found (Table 3). The pancreatic transection was more frequently on the left aortic border in the MIDP group (*p* = 0.005).

**Table 3:** Preoperative, intraoperative, and pathological data after propensity score matching

Study Population N° = 120				
	Total n° (%)	Open DP 80 (67%)	MI-DP 40 (33%)	*p*-value
*Preoperative*				
Age (years, DS)	74 ± 3	74 ± 4	73 ± 3	0.127
Sex (Female)	64 (53%)	43 (53%)	21 (54%)	0.547
BMI (Kg/m^2^, DS)	25 ± 4	25 ± 4	25 ± 4	0.720
Previous abdominal surgery	65 (54%)	45 (55%)	20 (51%)	0.403
ASA score > III	29 (24%)	22 (27%)	7 (18%)	0.191
Charlson Age > 4	34 (28%)	26 (32%)	8 (21%)	0.134
Neoadjuvant therapy	19 (16%)	14 (17%)	5 (13%)	0.367
Frailty index > 0	87 (73%)	59 (73%)	28 (72%)	0.534
*Intraoperative*				
Spleen preserving	11 (9%)	5 (6%)	6 (15%)	0.099
Additional resection	30 (25%)	23 (28%)	7 (18%)	0.156
Vascular resection	15 (13%)	12 (15%)	3 (8%)	0.212
Transection level				**0.005**
Pancreatic neck	111 (93%)	78 (98%)	33 (82%)	
Left aortic border	9 (7%)	2 (2%)	7 (18%)	
Management Stump				0.099
Stapler	64 (53%)	47 (58%)	17 (44%)	
Ultrasonic scalpel	56 (47%)	33 (42%)	23 (56%)	
Duration of Surgery (minutes, DS)	261 ± 92	252 ± 86	261 ± 84	0.166
EBL (cc, IQR)	250 [100–400]	265 [100–400]	200 [150–300]	0.101
*Pathological*				
Final Pathology, No. (%)				0.071
PDAC	60 (50%)	43 (53%)	16 (43%)	
pNET	25 (21%)	9 (12%)	16 (40%)	
IPMN	15 (13%)	12 (15%)	3 (7%)	
MCN/SCN	8 (7%)	5 (6%)	3 (7%)	
Other	12 (9%)	11 (14%)	1 (3%)	
Tumor Size (mm, IQR)	27 [20–40]	30 [20–40]	24 [18–33]	0.238
Harvest Lymph nodes (IQR)	25 [16–34]	26 [19–39]	25 [13–32]	0.054
R0 Status	103 (86%)	70 (88%)	33 (83%)	0.473

Bold value indicates statistical significance at the *p* < 0.05 level

*BMI* Body mass index; *ASA* American society of Anesthesiology; *EBL* Estimated blood loss; *PDAC* Pancreatic ductal adenocarcinoma; *pNET* pancreatic neuroendocrine tumor; *IPMN* Intraductal pancreatic mucinous neoplasm; *MCN* Mucinous cystic neoplasm; *SCN* Serous cystic neoplasm

None of the postoperative variables considered stratified differently from a statistical standpoint between the two groups (Table 4). Remarkably, the ODP group still had a higher mean length of stay than the MIDP (16 vs. 10 days, respectively, *p* = 0.046).

**Table 4:** Postoperative outcomes after propensity score matching

Study Population N° = 120				
	Total n° (%)	Open DP 80 (67%)	MI-DP 40 (33%)	*p*-value
Any complications	71 (59%)	48 (59%)	23 (59%)	0.565
POPF	25 (21%)	16 (20%)	9 (24%)	0.396
Abdominal collections	46 (38%)	30 (37%)	16 (41%)	0.411
DGE	4 (3%)	4 (5%)	0 (0%)	0.203
PPH	16 (13%)	11 (14%)	5 (13%)	0.578
SSI	11 (9%)	8 (10%)	3 (8%)	0.493
Pulmonary complications	29 (24%)	20 (25%)	9 (23%)	0.519
Cardiovascular complications	10 (8%)	9 (11%)	1 (3%)	0.104
Postoperative Confusion	3 (3%)	3 (4%)	0 (0%)	0.304
ICU Admission	8 (7%)	6 (7%)	2 (5%)	0.486
Clavien-Dindo > 3	16 (13%)	12 (15%)	4 (10%)	0.353
Length of Stay (days, DS)	13 ± 14	16 ± 20	10 ± 7	**0.046**
Reoperations	7 (6%)	5 (6%)	2 (5%)	0.590
Readmissions	10 (8%)	6 (8%)	4 (10%)	0.425
Mortality	1 (1%)	1 (1%)	0 (0%)	0.672

Bold value indicates statistical significance at the *p* < 0.05 level

*POPF* Postoperative pancreatic fistula; *DGE* Delayed gastric empty; *PPH* Post pancreatectomy hemorrhage; *ICU* Intensive care unit

### Baseline, surgical, and pathology results and postoperative outcome according to mFI

Among the baseline features, BMI and ASA score were higher in severely frail patients (*p* = 0.001 and *p* <  0.001, respectively), as shown in Table 5. Surgery’s duration increased as the severity of frailty did (*p* = 0.013). No differences were found in pathology data among the three groups.

**Table 5:** Stratification of the study population according to the modified Frailty Index

Study Population N° = 204				
	No Frail 54 (26%)	Mildly Frail 125 (26%)	Severely Frail 25 (74%)	*p*-value
*Preoperative data*				
Age (years, DS)	75 ± 4	75 ± 4	75 ± 4	0.897
Sex (Female)	33 (61%)	65 (52%)	10 (40%)	0.205
BMI (Kg/m^2^, DS)	23 ± 3	25 ± 4	26 ± 4	**0.001**
Previous abdominal surgery	33 (61%)	66 (53%)	15 (60%)	0.535
ASA score > III	7 (13%)	35 (28%)	19 (76%)	** <0.001**
Neoadjuvant therapy	5 (9%)	19 (15%)	6 (24%)	0.221
*Intraoperative data*				
Spleen preserving	3 (6%)	7 (6%)	1 (4%)	0.947
Additional resections	17 (32%)	38 (30%)	13 (52%)	0.106
Vascular resections	9 (17%)	17 (14%)	5 (20%)	0.675
Transection level				0.387
Pancreatic neck	44 (82%)	101 (81%)	20 (80%)	
GDA level	5 (9%)	20 (16%)	4 (16%)	
Left aortic border	5 (9%)	4 (3%)	1 (4%)	
Management Stump				0.924
Stapler	21 (40%)	52 (45%)	12 (50%)	
Ultrasonic scalpel	18 (34%)	39 (33%)	7 (29%)	
Handsewn	14 (26%)	26 (22%)	5 (21%)	
Duration of Surgery (minutes, DS)	224 ± 64	271 ± 93	297 ± 101	**0.013**
EBL (cc, IQR)	200 [100–350]	250 [200–320]	260 [220–355]	0.565
*Pathological data*				
Final Pathology, No. (%)				0.414
PDAC	31 (58%)	71 (57%)	17 (68%)	
pNET	12 (22%)	17 (14%)	4 (16%)	
IPMN	5 (9%)	19 (15%)	3 (12%)	
MCN/SCN	1 (2%)	10 (8%)	0 (0%)	
Other	5 (9%)	8 (6%)	1 (4%)	
Tumor Size (mm, IQR)	30 [22–50]	25 [20–40]	28 [20–45]	0.799

Bold values indicate statistical significance at the *p* < 0.05 level

*BMI* Body mass index; *ASA* American society of Anesthesiology; *EBL* Estimated blood loss; *PDAC* Pancreatic ductal adenocarcinoma; *pNET* pancreatic neuroendocrine tumor; *IPMN* Intraductal pancreatic mucinous neoplasm; *MCN* Mucinous cystic neoplasm; *SCN* Serous cystic neoplasm

Increasing and statistically significant rates of many complications were found among the three groups, where the severely frail patients worsen, compared to mildly frail and no frail ones (Table 6). In particular, this refers to any complication (76% vs. 60% vs. 44%, *p* = 0.022), abdominal collection (52% vs. 37% vs. 20%, *p* = 0.014), pulmonary complication (54% vs. 24% vs. 17%, *p* = 0.001), postoperative confusion (12% vs. 3% vs. 2%, *p* = 0.047), and Clavien-Dindo severity ≥3 events (24% vs. 14% vs. 6%, *p* = 0.036). The length of stay was significantly longer in severely frail patients (16 vs. 13 vs. 12 days, *p* = 0.018).

**Table 6:** Postoperative outcomes of the series stratified according to the modified Frailty Index

Study Population N° = 204				
	No Frailty 54 (26%)	Mild Frailty 125 (26%)	Severe Frailty 25 (74%)	*p*-value
Any complications	24 (44%)	75 (60%)	19 (76%)	**0.022**
POPF	7 (13%)	27 (22%)	4 (16%)	0.824
Abdominal collections	11 (20%)	46 (37%)	13 (52%)	**0.014**
DGE	0 (0%)	4 (3%)	1 (4%)	0.387
PPH	5 (9%)	16 (13%)	4 (16%)	0.667
SSI	5 (9%)	10 (8%)	3 (12%)	0.806
Pulmonary complications	9 (17%)	30 (24%)	14 (54%)	**0.001**
Cardiovascular complications	1 (2%)	14 (11%)	3 (12%)	0.108
Postoperative Confusion	1 (2%)	4 (3%)	3 (12%)	**0.047**
ICU Admission	2 (4%)	12 (10%)	3 (12%)	0.327
Clavien-Dindo > 3	3 (6%)	18 (14%)	6 (24%)	**0.036**
Length of Stay (days, DS)	12 ± 6	13 ± 9	16 ± 12	**0.018**
Reoperations	2 (4%)	7 (6%)	2 (8%)	0.724
Readmissions	4 (7%)	7 (6%)	3 (12%)	0.512
Mortality	0 (0%)	2 (2%)	0 (0%)	0.525

Bold values indicate statistical significance at the *p* < 0.05 level

*POPF* Postoperative pancreatic fistula; *DGE* Delayed gastric empty; *PPH* Post pancreatectomy hemorrhage; *ICU* Intensive care unit

## Discussion

Reduced functional reserve and stress response and high prevalence of comorbidities are typical features of the elderly. Thus, this population of patients may benefit the most from minimally invasive surgery. However, only four reports compared surgical outcomes of MIDP versus ODP in elderly patients [12–15]. All studies concluded that MIDP is at least as safe as ODP. These results were pooled in a meta-analysis that reported an overall benefit for MIDP over ODP regarding length of stay and intraoperative blood loss [16]. The results of this study corroborate these previous findings, namely that MIDP in elderly patients can be safely performed, as no differences were found in terms of postoperative outcomes. In particular, major complications, surgical and non-surgical complications (e.g., cardiopulmonary events) occurred homogeneously between the index and control groups, and the length of stay was similar. It must be noted that this study's findings are the result of a careful selection of cases to treat with MIDP, deriving from proper preoperative evaluation and collegial discussion.

Noteworthily, the duration of hospitalization of MIDP patients (median 8, IQR 7–12) was lower than the one institutionally reported for MIDP (10 days, IQR 6–10), deriving from a cohort of 103 patients with a mean age of 52 years [IQR 40–62]) [37]. This finding gain strength considering that all patients are discharged home in our institution rather than going to health care facilities. So, a patient goes home when the spouse or the relatives/caregivers (usually the children) are ready to accommodate him, which may take longer when the patient is elderly.

Despite desirable, a preoperative geriatric assessment may not be feasible for each elder patient. Thus, the possibility to use the mFI to identify elderly patients at higher risk of postoperative complications is an interesting opportunity. After applying the mFI to the whole cohort of patients ≥70 years submitted to DP, the baseline features analysis showed that BMI was proportionally higher for mildly and severely frail patients than non-frail ones. They were also more frequently classified as ASA ≥3, probably due to more systemic diseases (e.g., diabetes, hypertension, cardiovascular problems). The duration of surgery increased proportionally to the grade of frailty. We do not have an explanation for this. This may be due to the surgeon’s attitude toward performing a more precise and safe surgery in the presence of a severely frail patient, which carries serious comorbidities. However, this is just speculation.

As already reported [22, 38], the mFI predicted some postoperative complications and, in general, a worse postoperative course, as demonstrated by a longer length of stay. In detail, higher rates of any complication, abdominal collection, and Clavien-Dindo ≥3 complications were found in mild and severely frail patients, compared to non-frail ones. Noteworthily, the mFI also predicted non-surgical postoperative complications, such as postoperative confusion and pulmonary complications. These results show that surgical and non-surgical risk prediction may be obtained using an easy tool such as the mFI.

It must be noted that, interestingly, after DP, some postoperative complications, such as pancreatic fistula, post-pancreatectomy hemorrhage, and the reoperation rate, are not superior in frail patients. We believe that they are hardly favored by the cardiovascular comorbidities and the poor functional reserve that may be present in a frail patient. Rather, they are the result of properly pancreas- and surgery-specific factors (e.g., pancreatic texture, transection technique, level of transection, and drain management policy).

In general, our findings demonstrate that MIDP is not inferior to ODP in selected elderly patients, and that fragile elderly patients are at higher risk for postoperative complications after DP, with a proportionate increase. In the absence of a geriatric assessment, the application of the mFI may be useful to identify patients to whom dedicate tailored perioperative management to improve the postoperative outcome (e.g., nutritional assessment and intervention, pre-habilitation, routine access to intensive care unit after surgery). We deliberately choose not to include the mFI among the crucial variables in the PSM since previous literature on mFI and distal pancreatectomy was not robust enough. The present results give consistency to mFI as a variable to include in the baseline assessment of elderly patients receiving DP.

This manuscript's strength is that for the first time, the comparison between MIDP and ODP in elderly patients was performed through a propensity score matching to reduce the selection bias. This study is also the first to apply the mFI to a single-center, homogeneous cohort of elderly patients undergoing DP (the report by Konstantinidis et al. [22] is a registry-based study). Some limitations may flaw the results and the considerations: (i) the sample size of elderly who underwent MIDP is small; (ii) a selection bias (the percentage of elderly submitted to MIDP is approximately only 20 percent of the total of elderly underwent DP during the study period).

To conclude, MIDP in elderly patients seems to be safe in experienced hands and high-volume centers. Fragile (mFI > 0) elderly patients undergoing DP are more prone to experience postoperative complications, longer hospitalization, and worse postoperative course as the mFI increases. Age *per sé* is not an expression of poor functional reserve or difficult recovery after surgery. Age and frailty, instead, can be so.

## Supplementary Information

Below is the link to the electronic supplementary material.

Imbalances between MIDP and ODP groups before and after propensity score weighting. This figure represents the absolute standardized differences, showing imbalances of patients’ baseline characteristics between MIDP and ODP groups before and after propensity score weighting. A standardized difference <0.1 (<10%) is considered ideal (excellent covariate balance), <0.2 (20%) is considered acceptable. Red circle symbol: without weighting. Blue circle symbol: using the inverse of the propensity score as a weight (JPG 25 KB)
