# Supplementary figures and images for: Modified Frailty Index to Assess Risk in Elderly Patients Undergoing Distal Pancreatectomy: A Retrospective Single-Center Study
Source: World J Surg. 2022 Jan 13;46(4):891–900. doi: 10.1007/s00268-021-06436-2 (PMC8885554; doi:10.1007/s00268-021-06436-2)

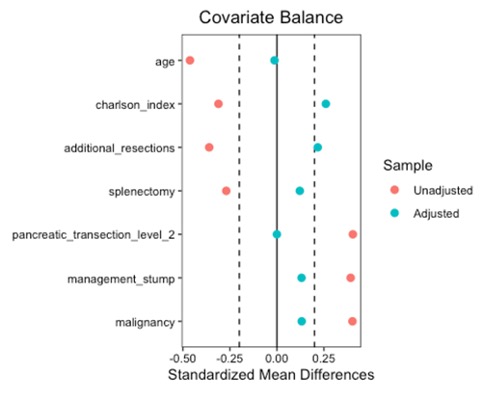

Supplement: Supplementary file 1 — Imbalances between MIDP and ODP groups before and after propensity score weighting. This figure represents the absolute standardized differences, showing imbalances of patients’ baseline characteristics between MIDP and ODP groups before and after propensity score weighting. A standardized difference <0.1 (<10%) is considered ideal (excellent covariate balance), <0.2 (20%) is considered acceptable. Red circle symbol: without weighting. Blue circle symbol: using the inverse of the propensity score as a weight (JPG 25 KB) [file 268_2021_6436_MOESM1_ESM.jpg]
